# Inhibition of microRNA-183 expression resists human umbilical vascular endothelial cells injury by upregulating expression of IRS1

**DOI:** 10.1080/10717544.2019.1628117

**Published:** 2019-06-18

**Authors:** Yingying Zhang, Yefei Zhan, Dandan Liu, Bo Yu

**Affiliations:** a Department of Cardiology, 2nd Affiliated Hospital of Harbin Medical University, and the Key Laboratory of Myocardial Ischemia, Chinese Ministry of Education, Harbin, People’s Republic of China;; b Department of Intensive Care Unit, Ningbo No 2 Hospital, Ningbo, People’s Republic of China

**Keywords:** MicroRNA-183, IRS1, human umbilical vascular endothelial cells, oxidized low-density lipoprotein, cell injury

## Abstract

Our study aims to investigate the effect of microRNA-183 (miR-183) on human umbilical vascular endothelial cells (HUVECs) injury by targeting IRS1. HUVECs injury was induced by oxidized low-density lipoprotein (ox-LDL). HUVECs were grouped so as to explore the role of ox-LDL and miR-183 in HUVECs injury, with the expression of miR-183 and IRS1 detected. Additionally, the related factors of oxidative stress and inflammation, as well as angiogenesis ability, proliferation, cell cycle, apoptosis, invasion, and migration abilities were also measured. Ox-LDL treatment could decrease the activity of HUVECs, increase the level of oxidative stress and inflammation, and induce the HUVECs injury. miR-183 could inhibit the expression of IRS1. The inhibition of miR-183 expression in ox-LDL-induced HUVECs injury could enhance cell activity, inhibit inflammatory level, and thus resist cell injury. Low expression of IRS1 could reverse the inhibition of miR-183 on HUVECs injury. This study highlights that inhibition of miR-183 expression may resist HUVECs injury by upregulating expression of IRS1.

## Introduction

Endothelial cells (ECs) exert a host of biological functions, such as angiogenesis, vascular permeability, and endothelial hyperpermeability (Li et al., 2017). ECs have abnormal structural features in tumors, such as fenestrations and widened intercellular junctions (Dudley, 2012), which forms hyperpermeable and angiogenic vessels (Omori et al., 2018). Injury and/or denudation of ECs can trigger leukocytes attachment to the subendothelial region and also induce transendothelial migration of cells, initiating atherosclerosis (Yamawaki & Iwai, 2006). Angiogenesis is known as a biological process which generates new blood vessels originating from existing vascular endothelial cells (VECs), so as to deliver oxygen and nutrients to various organs and tissues (Carmeliet, 2005). It has recently been reported that oxidized low-density lipoprotein (ox-LDL) plays a significant role in early inflammatory processes, which might induce atherosclerotic lesions (Zhang & Jiang, 2016). Besides, ox‑LDL, in the process of atherosclerotic lesion formation, is capable of promoting the initiation of monocyte invasion (Mitra et al., 2011).

MicroRNAs (miRNAs) are small and noncoding RNAs which post-transcriptionally modulate gene expression, and they also play a role in keeping normal cellular functions (Najafi-Shoushtari et al., 2010; Ramirez et al., 2013). Deregulation of miRNA expression results in the onset of different types of diseases, including cancer, sarcomas, and hematologic tumors (Lu et al., 2005; Calin et al., 2008; Mendell, 2008; Subramanian et al., 2008; Sarver et al., 2010). miR-183 is a member of an miRNA family consisting of three members, namely, miR-96, miR-182, and miR-183, which is located on human chromosome 7 (Pierce et al., 2008). Previously published data indicated that significant or mild upregulation of miR-183 was found in different types of cancers, including lung cancer, bladder cancer, colon cancer, prostate cancer, and hepatocellular carcinoma cells (Bandres et al., 2006; Sarver et al., 2009; Yamada et al., 2011; Liang et al., 2013; Tsuchiyama et al., 2013; Xu et al., 2014). A previous study has reported that the repression of miRNA is based on the varying conditions of specific cellular targets (Doench & Sharp, 2004). Insulin receptor substrate 1(IRS1) is a member of insulin receptor substrates (IRSs) family, which is able to integrate and coordinate hormone, cytokine, and growth factor signal (Dearth et al., 2006). Besides, IRS1 acts as a transforming ontogeny that could induce transformation and metastasis both in vitro and in vivo (Chan & Lee, 2008). It has been demonstrated that knockdown of MEG3 is able to alleviate hypoxia-induced H9c2 cell injury through miR-183-mediated suppression of p27 by activating the PI3K/AKT/FOXO3a pathway (Gong et al., 2018). Based on this, we performed this study to figure out the role of miR-183 in HUVECs injury by targeting IRS1, so as to find out a new therapeutic target for the treatment of this disease.

## Materials and methods

### Establishment of HUVECs injury model induced by oxidized low-density lipoprotein (ox-LDL)

HUVECs were cultured on Dulbecco’s modified Eagles Medium (DMEM) containing 10% fetal bovine serum (FBS). When with 90% of confluency, the cells were detached with trypsin and inoculated into a 96-well plate with 7 × 10^3^ cells per well. Next, the cells were incubated in an incubator of 5% CO_2_ at 37 °C for 24 h. The culture medium was absorbed and then cultured for 24 h with the final concentration of 50, 100, 150, and 200 μg/mL for 24 h, respectively.

### Cell counting kit-8 (CCK-8) assay

When the cells treated in each group reached about 80% confluence, the cells were rinsed with PBS twice. The cells were routinely trypsinized and pipetted into a single cell suspension. The cells were counted by a cell counter. The cells were cultured with a 96-well plate, and the culture medium was discarded at a specific time. After that, the fresh culture medium containing 10 μL CCK-8 reagent (Beyotime Biotechnology Co., Ltd., Shanghai, China) was added. The well plate was incubated in an incubator with CO2 for 2 h, and the optical density (OD) value at 450 nm wavelength was measured by a microplate reader (Bio-Rad, Laboratories, Hercules, CA, USA). Six parallel wells were set up in this experiment. Cell viability = [(experimental well OD450 value - blank well OD450 value)/(control well OD450 value - blank well OD450 value)] × 100%. The experiment was repeated three times.

### Cell grouping

Normal cultured HUVECs were used as blank group. HUVECs treated with 150 μg/mL ox-LDL for 24 h were used as ox-LDL group. HUVECs treated with 150 μg/mL ox-LDL transfected with the negative control (NC) sequence of miR-183 analogs were used as ox-LDL + mimics NC group. HUVECs treated with 150 μg/mL ox-LDL transfected with the sequence of miR-183 analogs were used as ox-LDL + miR-183 mimics group. HUVECs treated with 150 μg/mL ox-LDL transfected with the NC sequence of miR-183 inhibitor were used as ox-LDL + inhibitors NC group. HUVECs treated with 150 μg/mL ox-LDL transfected with the sequence of miR-183 inhibitor were used as ox-LDL + miR-183 inhibitors group. HUVECs treated with 150 μg/mL ox-LDL transfected with the sequence of miR-183 inhibitors and the NC sequence of IRS1 siRNA were used as ox-LDL + miR-183 inhibitors + siRNA-NC. HUVECs treated with 150 μg/mL ox-LDL transfected with the sequences of miR-183 inhibitors and IRS1 siRNA were used as ox-LDL + miR-183 inhibitors + IRS1 siRNA. The sequence is shown in Table 1.

**Table 1.** Transfection sequence.

Name	Sequence
miR-183 mimics	Forward: 5′-UAUGGCACUGGUAGAAUUCACU-3′
	Reverse: 5′-UGAAUUCUACCAGUGCCAUAUU-3′
miR-183 inhibitors	Forward: 5′-AGUGAAUUCUACCAGUGCCAUA-3′
	Reverse: 5′-UAUGGCACUGGUAGAAUUCACU-3′
mimics NC	Forward: 5′-UUCUCCGAACGUGUCACGUTT-3′
	Reverse: 5′-ACGUGACACGUUCGGAGAATT-3′
inhibitors NC	Forward: 5′-UUCUCCGAACGUGUCACGUTT-3′
	Reverse: 5′-ACGUGACACGUUCGGAGAATT-3′
IRS1 siRNA	Forward: 5′-TGTCAGTCTGTCGTCCAGTATTCAAGAGATACTGGACGACAGACTGACTTTTTTC-3′
	Reverse: 5′-TCGAGAAAAAAGTCAGTCTGTCGTCCAGTATCTCTTGAATACTGGACGACAGACTGACA-3′
IRS1 siRNA NC	Forward: 5′-TACAAGACCTAAGTGCACTGTTCAAGAGACAGTGCACTTAGGTCTTGTTTTTTTC-3′
	Reverse: 5′-TCGAGAAAAAAACAAGACCTAAGGCACTGTCTCTTGAACAGTGCACTTAGGTCTTGTA-3′

mir-183: microRNA-183; NC: negative control.

### Reverse transcription quantitative polymerase chain reaction (RT-qPCR)

MiRNeasy Mini Kit (Qiagen, Valencia, CA) was used to extract total RNA from cells in logarithmic growth phrase. The samples of 5 μL RNA were diluted 20 times with ultrapure water without RNA enzyme to read the OD values of RNA at 260 and 280 nm, and the concentration and purity of RNA were determined. The ratio of OD260/OD280 was between 1.7 and 2.1, which indicated that the purity was high, which could meet the need of further experiments. The cDNA template was synthesized by reverse transcription reaction with a PCR amplification instrument, and RT-qPCR experiment was carried out by ABI7500 quantitative PCR instrument. The reaction conditions were as follows: pre-denaturation at 95 °C for 3 min, with a total of 30 cycles of denaturation at 94 °C for 30 s, annealing at 55 °C for 30 s, and extension at 72 °C for 30 s. The primers used in the reaction are shown in Table 2 with β-actin as an internal reference, and each sample repeated 3 times. The results of PCR were analyzed by OpticonMonitor3 software (Bio-Rad, Laboratories, Hercules, CA). The data were analyzed by 2^−ΔΔCt^ method.

**Table 2.** Primer sequence for RT-qPCR.

Name	Sequence
miR-183	Forward: 5′-ACACTCCAGCTGGGTATGGCACTGGTAGAA-3′
	Reverse: 5′-CTCAACTGGTGTCGTGGAGTCGGCAATTCAGTTGAGA-3′
18S-rRNA	Forward: 5′-GTGGTGTTGAGGAAAGCAGACA-3′
	Reverse: 5′-TGATCACACGTTCCACCTCATC-3′
IRS1	Forward: 5′-ATGTCGCCAGTGGGAGTT-3′
	Reverse: 5′-CTTCGGCAGTTGCGGTATA-3′
β-actin	Forward: 5′-GGCACCACACCTTCTACAATG-3′
	Reverse: 5′-GGGGTGTTGAAGGTCTCAAAC-3′

mir-183: microRNA-183.

### Western blot analysis

Protein lysate (NanJing KeyGen Biotech Co., Ltd, Nanjing, China) added in cells of logarithmic growth period was used to extract total protein. Bradford method (NanJing KeyGen Biotech Co., Ltd, Nanjing, China) was used for protein quantification. The total protein (50 μg) was transferred to polyvinylidene difluoride (PVDF) membrane (Millipore), after 12% sodium dodecyl sulfate polyacrylamide gel electrophoresis (SDS-PAGE). The membrane was blocked with 5% dried skimmed milk at 37 °C for 1 h, and then supplemented with IRS1 and β-actin monoclonal antibodies (1:1000; Abcam, UK) and incubated at 4 °C overnight. The membrane was washed with PBST three times, each time for 5 min, and then added with horseradish peroxidase-labeled secondary antibody (Abcam, UK; 1:4000) and incubated at room temperature for 1 h. The positive contact reaction between the working fluid of electrogenerated chemiluminescence (ECL) solution and the PVDF membrane was 3–5 min. The solution of ECL was filtered out and developed into the dark room. The gray value was calculated by Image J image analysis software. The ratio of the gray value of the target band to the internal reference band β-actin was taken as the relative expression level of protein. Each experiment was repeated three times.

### Double luciferase reporter gene assay

The target gene statistics of miR-183 were carried out by using database TargetScan. IRS1 was preliminary selected as the direct target gene of miR-183. After inoculating cardiacmyocytes at the well plate, the total length of the 3′ UTR region of the wild-type (wt) IRS1 gene was cloned and amplified when the cell confluency reached about 70%. The PCR product was cloned into pMIR-REPOR™ Luciferase (Promega Corporation, Madison, WI) vector and named as pMI/IRS1-wt vector. The pMIR/IRS1-mutant type (mut) vector was constructed by site-directed mutation of the binding site between miR-183 and target gene, which was predicted by bioinformatics information. The vector was used to adjust cell number and transfection efficiency by using phRL-TK vector expressing Renilla luciferase (TaKaRa company) as internal reference. Two reporter gene vectors (pMIR/IRS1-wt and pMIR/IRS1-mut) and miR-183 mimic and their NC sequences (miR-183 NC) were co-transfected into cardiomyocytes to detect the activity of double luciferase according to the method provided by Promega Corporation (Madison, WI).

### Reactive oxygen species (ROS) detection

Cell precipitation was collected after trypsin digestion and then centrifuged at 2000 rpm for 4 min. A 600 µL serum-free DMEM medium (the ratio of DCFH-DA was 1:1000 and the working fluid concentration of DCFH-DA was 10 µM) was used to incubate for 20 min without light. During incubation, the samples are mixed upside down every 5 min to ensure full binding between the probe and the cells. After incubation, the cell precipitation was collected after centrifugation for 4 min at 2000 rpm and then suspended and centrifuged on serum-free DMEM medium. This step was repeated 3 times to fully remove unbonded DCHF-DA dyes. After the last centrifugation, the cells were precipitated with 200–300 µL serum-free DMEM culture medium. The fluorescence intensity of ROS was measured by BD Accuri C6 flow cytometry (BD), and the level of ROS was expressed by fluorescence intensity.

### Determination of malonaldehyde (MDA) and superoxide dismutase (SOD) content

Cells detached by trypsin were placed in a centrifuge tube and centrifuged at a speed of 3000 r/min for 15 min. The supernatant was put it in a −20 °C cryogenic refrigerator for detection. The supernatant (100 μL) was absorbed, and the activity of MDA and SOD was measured by an automatic microplate reader (BECKMAN-CoμLter Inc.) and the content of MDA and SOD was detected by the instructions of kits (Shanghai Haling Biotechnology Co., Ltd., Shanghai, China). The experiment was repeated three times.

### Enzyme-linked immunosorbent assay (ELISA)

After the cells were detached by trypsin, the cells were collected and precipitated by centrifugation. The procedures were in strict accordance with the instructions of the ELISA kit (EM008-48, Shanghai Genetimes Biotechnology Co., Ltd, Shanghai, China). The ELISA kit was balanced for 20 min at room temperature and the detergent was prepared. After the standard sample was dissolved, 100 μL of them was added to the reaction plate to make the standard curve. The sample to be detected was incubated with 100 μL in the reaction well at 37 °C for 90 min. After washing, 100 μL of biotinylated antibody working fluid was added to incubate at 37 °C for 60 min. After washing, 100 μL of enzyme binding reactants working fluid was added to incubate at 37 °C for 30 min. The plates were washed for 3 times and incubated with 100 μL at 37 °C for 15 min, followed by rapid addition of termination reaction. An automatic microplate reader (BioTek Synergy 2) was used to detect the OD value of each well at the wavelength of 450 nm within 3 min, and a curve was drawn based on the OD value. The experiment was repeated three times. The contents of TNF-α, IL-10, and IL-1β were measured and the results were analyzed.

### Lumen formation experiment

Using lumen formation experiment, the matrigel was evenly laid on the bottom of the 96-well plate according to 50 μL, and then placed in an incubator of 5% CO_2_ at 37 °C with a relative humidity of 95% for 2 h to solidify the matrigel. The cell density of each group was adjusted to 5 × 10^7^ L^−1^ with RPMI1640 serum-free medium. The cells were inoculated into 96-well plate with 100 μL in each well. The same amount of phosphate buffer saline (PBS) was added as the blank control group. Each group was divided into three multiple wells. After 8 h of conventional culture, the formation of lumen of each experimental group was observed under an inverted microscope. The experiment was repeated three times. The formation number and length of the lumen were obtained by IPP software.

### Flow cytometry

Propidium iodide (PI) staining: After 48 h of transfection, the cells were collected and fixed with −20 °C precooled 75% ice ethanol, and then placed in a 4 °C refrigerator overnight. After centrifugation, the cells were precipitated with cold PBS for 2 times to remove the fixed solution, and then added with RNaseA, and water-bathed devoid of light for 30 min. after that, the cells were stained with PI, and after mixing evenly, the cell cycle was recorded by flow cytometry and red fluorescence was used to detect cell cycle. The experiment was repeated three times.

AnnexinV/PI double staining: After 48 h of transfection, the cells were collected and the density was adjusted to 1 × 10^6^/mL. The cell suspension (0.5 mL) was taken into a centrifuge tube and added with 1.25 μL AnnexinV-FITC (NanJing KeyGen Biotech Co., Ltd, Nanjing, China), incubated at room temperature devoid of light for 15 min. Afterward, the cells were centrifuged at 1000 rpm for 5 min, with the supernatant discarded. The cells were gently suspended with 0.5 mL precooled binding buffer and added with 10 μL PI, followed by immediate detection and analysis by flow cytometry (BD). The right lower quadrant represented early apoptotic cells (ITC^+^/PI^−^), the right upper quadrant (Q2) was necrosis and late apoptotic cells (FITC^+^/PI^+^), the apoptosis rate = early apoptosis percentage (Q3)+late apoptosis percentage (Q2).

### Transwell assay

The matrigel (40 μL) dissolved at 4 °C was added to the precooled Transwell chamber and incubated in a cell incubator for 1 h to make matrigel gelatinized. After 48 h of transfection, the cell concentration was adjusted to 1 × 10^5^/100 μL with serum-free medium. The cell suspensions (100 μL) were added to the apical chamber of Transwell (BD) in the 24-well plate. The basolateral chamber was incubated with 500 μL medium containing 10% FBS and incubated in a 5% CO_2_ incubator at 37 °C for 48 h. The chamber was removed and the cells in the apical chamber were scrubbed with cotton swab, fixed with 4% paraformaldehyde for 15 min, washed with PBS one time, stained with crystal violet for 10 min, and washed with PBS one time again. The upper, lower, left, right, and middle visual fields was selected and counted under a high-power microscopy (Olympus, Japan), and the number of cells passing through the wells was calculated to detect cell invasion (with the number of cells in the blank group as a reference, the ratio of the number of cells in each group to the number of cells in blank group was calculated). The same step experiment was carried out in Transwell chamber without matrigel to measure cell migration ability.

### Statistical analysis

All the data were analyzed by SPSS 21.0 software (IBM Corp, Armonk, NY). The Kolmogorov–Smirnov test verified that the data in this study had a normal distribution. Measurement data were expressed as mean ± standard deviation. The *t* test was used for the comparison between the two groups, and one-way analysis of variance (ANOVA) was used for the comparison among three or more groups. The Fisher’s least significant difference *t* test (LSD-*t*) was employed for pairwise comparison. The level of significance was *p* < .05.

## Results

### Effects of ox-LDL on viability, expression of related factors, and biological function of HUVECs cells

**Figure 1.**
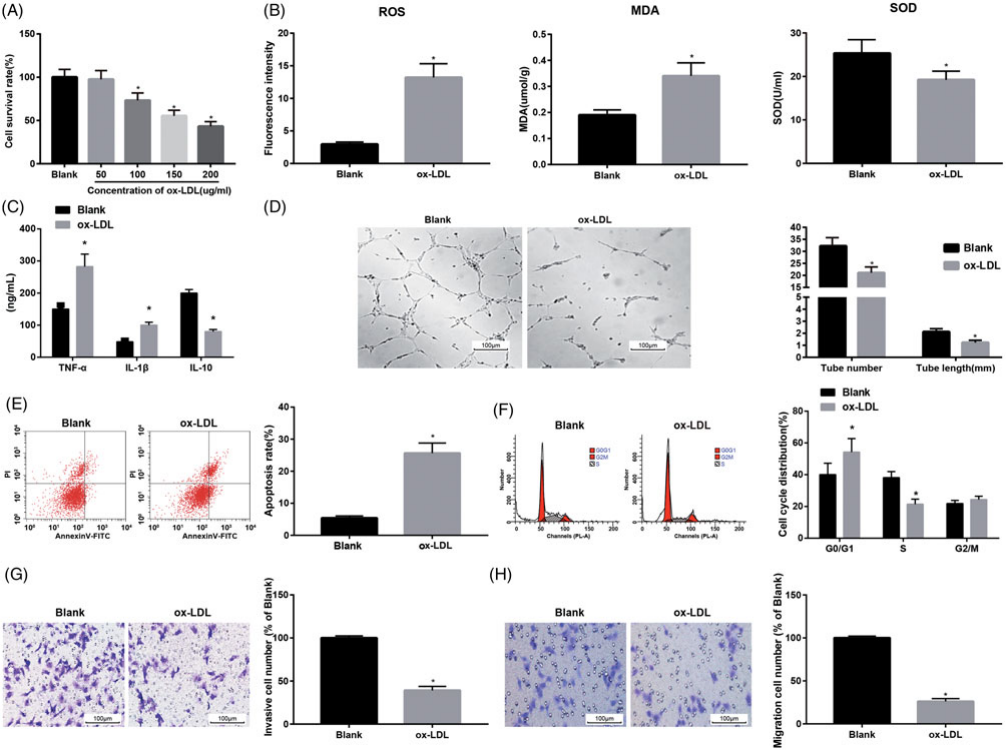
Ox-LDL treatment decreases the activity of HUVECs and increases the level of oxidative stress and inflammation. (A) Effect of ox-LDL treatment on proliferation of HUVECs by CCK-8 assay. (B) Expression of oxidative stress-related factors in HUVECs. (C) Expression of inflammation-related factors in HUVECs. (D) Experimental detection of HUVECs lumen formation and analysis of the number of lumen formation and length of lumen. (E) Analysis of apoptosis and apoptosis rate in HUVECs cells by flow cytometry. (F) Analysis of HUVECs cell cycle and cell cycle ratio by flow cytometry. (G) Analysis of migration ability and cell migration number of HUVECs detected by Transwell assay. (H) Analysis of invasion ability and cell invasion number of HUVECs detected by Transwell assay; *p < .05 versus the blank group.

Cell proliferation of HUVECs cells was detected by CCK-8 assay (Figure 1(A)). The results suggested that the activity of HUVECs treated with 50 μg/mL ox-LDL for 24 h showed little change, while in contrast to the blank group, the activity of HUVECs treated with 100, 150, and 200 μg/mL ox-LDL for 24 h decreased to 73.4%, 55.32%, and 43.27%, respectively, which showed a concentration-dependent relationship. In this study, HUVECs treated with150 μg/mL ox-LDL for 24 h was selected for the follow-up treatment. Meanwhile, the results of oxidative stress detection demonstrated that compared with the blank group, the levels of ROS and MDA in the ox-LDL group were increased while that of SOD was decreased (all *p* < .05; Figure 1(B)). Compared with the blank group, the levels of TNF-α and IL-1β in the ox-LDL group were upregulated, and the level of anti-inflammatory factor IL-10 was downregulated (all *p* < .05; Figure 1(C)). Besides, angiogenesis was detected by lumen formation experiment (Figure 1(D)), and the findings suggested that the number of lumen formation and the length of lumen were decreased in the ox-LDL group in comparison to the blank group. The results of flow cytometry showed that the apoptosis rate was increased and more cells were arrested at G0/G1 phrase while less in S phrase in the ox-LDL group compared with that in the blank group (Figure 1(E,F)). Furthermore, the results of Transwell assay (Figure 1(G,H)) suggested that in contrast to the blank group, the number of invasive cells and migrated cells was decreased in the ox-LDL group. The results showed that ox-LDL treatment could decrease the activity of HUVECs, increase the level of oxidative stress and inflammation, and induce HUVECs injury.

### Targeted inhibition of IRS1 expression by miR-183

**Figure 2.**
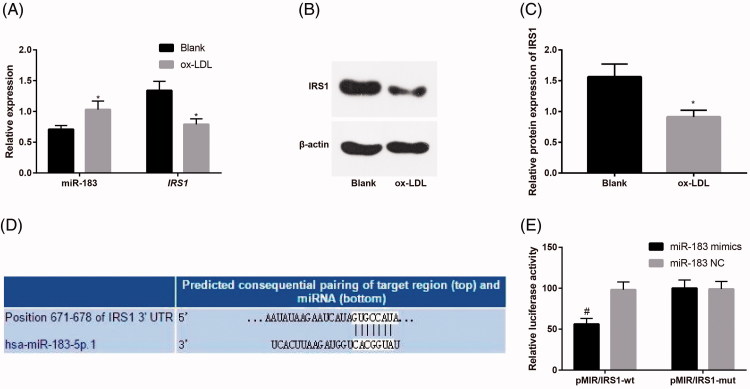
Interaction between miR-183 and IRS1. (A) Detection of miR-183 and IRS1 mRNA expression in HUVECs by RT-qPCR. (B) Protein bands of IRS1 in HUVECs. (C) Detection of IRS1 protein expression in HUVECs by western blot analysis. (D) TargetScan Database predicts that IRS1 is the target gene of miR-183. (E) Luciferase activity in each group by double luciferase activity determination; *p < .05 versus the blank group; #*p* < .05 versus the miR-183 NC group.

The results of RT-qPCR and western blot analysis indicated that in comparison to the blank group, the miR-183 expression was increased and the expression of IRS1 mRNA and protein was decreased in the ox-LDL group (all *p* < .05; Figure 2(A–C)). By searching the TargetScan database, we found that IRS1 was a potential target gene of miR-183 (Figure 2(D)). The results of double luciferase reporter gene assay indicated that the luciferase activity of pMIR/IRS1-wt in the miR-183 mimics group was significantly lower than that of the miR-183 NC group (*p* < .05). No significant difference was found in the luciferase activity of pMIR/IRS1-mut in the miR-183 mimics group relative to that of the miR-183 NC group (*p* > .05; Figure 2(E)). The results showed that miR-183 could inhibit the expression of IRS1.

### Inhibition of miR-183 resists ox-LDL-induced HUVEC injury

**Figure 3.**
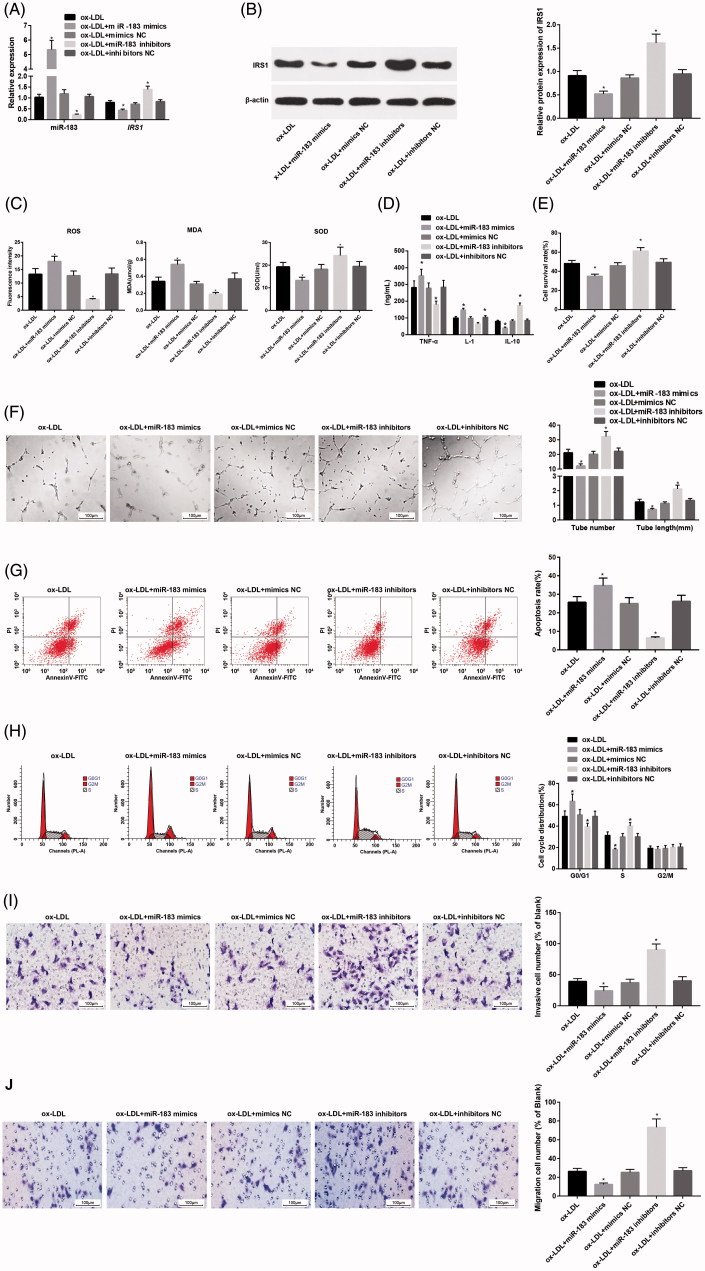
Effect of miR-183 on HUVECs injury induced by ox-LDL. (A) Detection of miR-183 and IRS1 mRNA expression in HUVECs by RT-qPCR. (B) Protein bands of IRS1 in HUVECs and detection of IES1 protein expression in HUVECs by western blot analysis. (C) Expression of oxidative stress related factors in HUVECs. (D) Expression of inflammation related factors in HUVECs. (E) Detection of cell survival rate of HUVECs by CCK-8 assay. (F) Experimental detection of HUVECs lumen formation and analysis of the number of lumen formation and length of lumen. (G) Analysis of apoptosis and apoptosis rate in HUVECs cells by flow cytometry. (H) Analysis of HUVECs cell cycle and cell cycle ratio by flow cytometry. (I) Analysis of migration ability and cell migration number of HUVECs detected by Transwell assay. (J) Analysis of invasion ability and cell invasion number of HUVECs detected by Transwell assay; *p < .05 versus the ox-LDL + miR-183 inhibitors + siRNA-NC group.

The results of RT-qPCR and western blot analysis indicated that in comparison to the ox-LDL group, the miR-183 expression was increased and the expression of IRS1 mRNA and protein was decreased in the ox-LDL + miR-183 mimics group, while the miR-183 expression was decreased and the expression of IRS1 mRNA and protein was increased in the ox-LDL + miR-183 inhibitors group (all *p* < .05; Figure 3(A–B)). Meanwhile, the results of oxidative stress detection demonstrated that compared with the ox-LDL group, the levels of ROS and MDA were increased while that of SOD was decreased in the ox-LDL + miR-183 mimics group, while the levels of ROS and MDA were decreased while that of SOD was increased in the ox-LDL + miR-183 inhibitors group (all *p* < .05; Figure 3(C)). Compared with the ox-LDL group, the levels of TNF-α and IL-1β were upregulated, and the level of anti-inflammatory factor IL-10 was downregulated in the ox-LDL + miR-183 mimics group, while the levels of TNF-α and IL-1β were downregulated, and the level of anti-inflammatory factor IL-10 was upregulated in the ox-LDL + miR-183 inhibitors group (all *p* < .05; Figure 3(D)). The cell proliferation of CCK-8 assay showed that the cell survival rate of the ox-LDL + miR-183 mimics group was lower than that of the ox-LDL group, and the cell survival rate of the ox-LDL + miR-183 inhibitors group was increased (both P < 0.05; Figure 3(E)). Besides, that the number of lumen formation and the length of lumen were decreased in the ox-LDL + miR-183 mimics group in comparison to the ox-LDL group, while the opposite results were found in the ox-LDL + miR-183 inhibitors group (Figure 3(F)). The results of flow cytometry showed that the apoptosis rate was increased and more cells were arrested at G0/G1 phrase while less in S phrase in the ox-LDL + miR-183 mimics group compared with that in the ox-LDL group, while the opposite results were found in the ox-LDL + miR-183 inhibitors group (Figure 3(G,H)). Furthermore, the results of Transwell assay suggested that in contrast to the ox-LDL group, the number of invasive cells and migrated cells were decreased in the ox-LDL + miR-183 mimics group, while the number of invasive cells and migrated cells was increased in the ox-LDL + miR-183 inhibitors group (all *p* < .05; Figure 3(I,J)). The ox-LDL + mimic NC group and ox-LDL + inhibitors NC group had no significant change in each index compared with the ox-LDL group. The results showed that the inhibition of miR-183 expression in ox-LDL-induced HUVECs injury could enhance cell activity, inhibit inflammatory level, and thus resist cell injury.

### Low expression of IRS1 reverses the inhibitory effect of miR-183 on HUVECs injury

**Figure 4.**
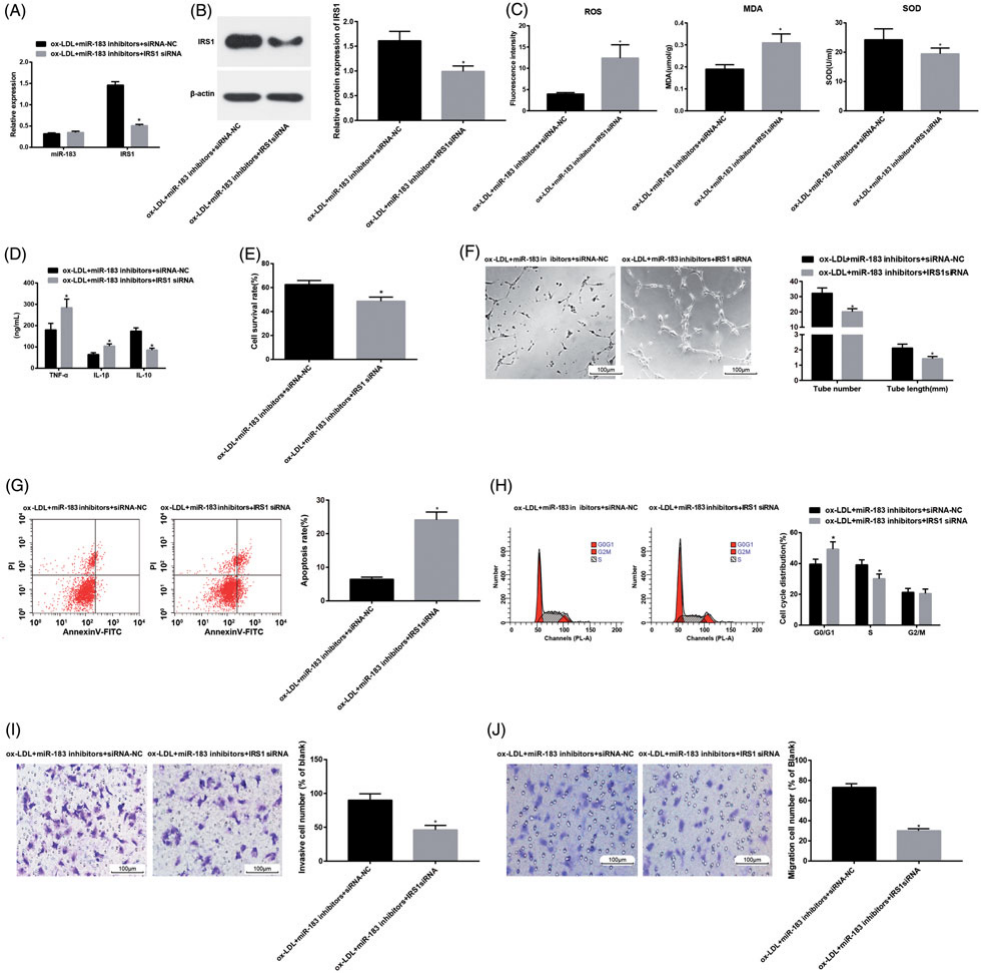
Effect of combined regulation of miR-183 and IRS1 on HUVECs injury induced by ox-LDL. (A) Detection of miR-183 and IRS1 mRNA expression in HUVECs by RT-qPCR. (B) Protein bands of IRS1 in HUVECs and detection of IES1 protein expression in HUVECs by western blot analysis. (C) Expression of oxidative stress related factors in HUVECs. (D) Expression of inflammation related factors in HUVECs. (E) Detection of cell survival rate of HUVECs by CCK-8 assay. (F) Experimental detection of HUVECs lumen formation and analysis of the number of lumen formation and length of lumen. (G) Analysis of apoptosis and apoptosis rate in HUVECs cells by flow cytometry. (H) Analysis of HUVECs cell cycle and cell cycle ratio by flow cytometry. (I) Analysis of migration ability and cell migration number of HUVECs detected by Transwell assay. (J) Analysis of invasion ability and cell invasion number of HUVECs detected by Transwell assay; *p < .05 versus the ox-LDL + miR-183 inhibitors + siRNA-NC group.

In contrast to the ox-LDL + miR-183 inhibitors + siRNA-NC group, in the ox-LDL + miR-183 inhibitors + IRS1 siRNA group, the miR-183 expression had no significant difference and the expression of IRS1 mRNA and protein was decreased (Figure 4(A–B)), the levels of ROS and MDA were increased while that of SOD was decreased (Figure 4(C)), the levels of TNF-α and IL-1β were upregulated, and the level of anti-inflammatory factor IL-10 was downregulated (Figure 4(D)), cell survival rate was decreased (Figure 4(E)), the number of lumen formation and the length of lumen were decreased (Figure 4(F)), the apoptosis rate was increased and more cells were arrested at G0/G1 phrase and less in S phrase (Figure 4(G,H)), the number of invasive cells and migrated cells was decreased (Figure 4(I,J)). The ox-LDL + miR-183 inhibitors + siRNA-NC had no significant change in each index compared with the ox-LDL + miR-183 inhibitors group. It was suggested that the low expression of IRS1 could reverse the inhibition of miR-183 on HUVECs injury.

## Discussion

Alterations in miRNAs expression have been discussed in all types of analyzed human tumors by different research groups, which were proposed to result in oncogenesis, by acting as either tumor suppressor genes or oncogenes (Esquela-Kerscher & Slack, 2006). Meanwhile, miRNAs are implicated in biological processes, including proliferation and differentiation, invasion, and apoptosis, which are all associated with tumorigenesis (Miska, 2005; Hwang & Mendell, 2006; Dykxhoorn, 2010). Evidence has demonstrated that miR-183 plays an essential role in tumorigenesis, which could serve as either an oncogene or a tumor-suppressor gene in different types of cancer (Li & Subramanian, 2010). However, no study has focused on the role of miR-183 in HUVECs injury. In view of this, this study aims to elucidate its potential role and collectively, the findings of this study suggested that inhibition of miR-183 expression may resist HUVECs injury by upregulating expression of IRS1.

One of the most significant findings revealed that ox-LDL treatment could decrease the activity of HUVECs, increase the level of oxidative stress and inflammation, and induce HUVECs injury. A previous study has shown that the plasma OxLDL level is able to act as a marker for vascular diseases, and it has been indicated the foam cell-associated accumulation of OxLDL is detected in human atherosclerotic lesions (Itabe et al., 2004). Additionally, oxLDL has been suggested to induce apoptosis in cultured vascular smooth muscle cells (VSMCs) (Okura et al., 2000). Furthermore, lectin-like oxidized low-density lipoprotein receptor-1 (LOX-1), acting as the main OxLDL in ECs, is reported to overexpressed in atherosclerotic lesions, which is participated in several cellular processes modulating the pathogenesis of atherosclerosis (Pirillo & Catapano, 2013).

Also, our study has proposed that miR-183 could inhibit the expression of IRS1. A previous study has shown PDCD4 might be a target gene of miR-183 in human hepatocellular carcinoma cells (Li et al., 2010). Additionally, several direct targets of miR-183 have also been proposed, such as ezrin in lung cancer cells, together with Dkk-3 and SMAD4 in prostate cancer cells (Wang et al., 2008; Ueno et al., 2013). Furthermore, miR-183 enables to promote proliferation and invasion in esophageal squamous cell carcinoma (ESCC) by targeting PDCD4 (Ren et al., 2014). In this study, for the purpose of determining the specific target gene that miR-183 controls in HUVECs injury, we analyzed the expression of IRS1 and found that miR-183 could inhibit the expression of IRS1, suggesting that IRS1 is a target gene of miR-183. Similar to our results, a study has found out that miR-195 inhabited tumor growth and angiogenesis by regulating IRS1 in breast cancer, suggesting that miR-195 might be considered as be a potential therapeutic target in the treatment of breast cancer (Wang et al., 2016).

Furthermore, our study demonstrated that the inhibition of miR-183 expression in ox-LDL-induced HUVECs injury could enhance cell activity, inhibit inflammatory level, and thus resist cell injury. Recently, miR-183 has been involved in the regulation of different stages of apoptotic and autophagy by modulating apoptosis and autophagy-related genes (Jian et al., 2011; Li et al., 2011; Fu et al., 2012). Specifically, the knockdown of miR-183 has been suggested to promote cell death in medullary thyroid cancer via regulation of certain tumor suppressive signaling pathways, implying that miR-183 may act as an attractive therapeutic target (Abraham et al., 2011). It has been reported the miR-183-mediated inhibition of migration and invasion, which shows that the miR-183 cluster miRNAs possess context-dependent functions (Li et al., 2014). Besides, the inhibition in migration and invasion of SW1990 pancreatic cancer cell resulted from miR-183 inhibition could be caused by the regulation of E-cadherin/N-cadherin expression (Lu et al., 2015).

To our knowledge, this study highlights that the inhibition of miR-183 expression in ox-LDL-induced HUVECs injury could enhance cell activity, inhibit inflammatory level, and thus resist cell injury. Additionally, low expression of IRS1 could reverse the inhibition of miR-183 on HUVECs injury. Our study might provide a pivotal avenue for further experiment with the aim to seek for a novel potential diagnostic and therapeutic target for the screening and treatment of HUVECs injury. However, further studies are still needed to fully comprehend the mechanisms of miR-183 and IRS1 in HUVECs injury.
